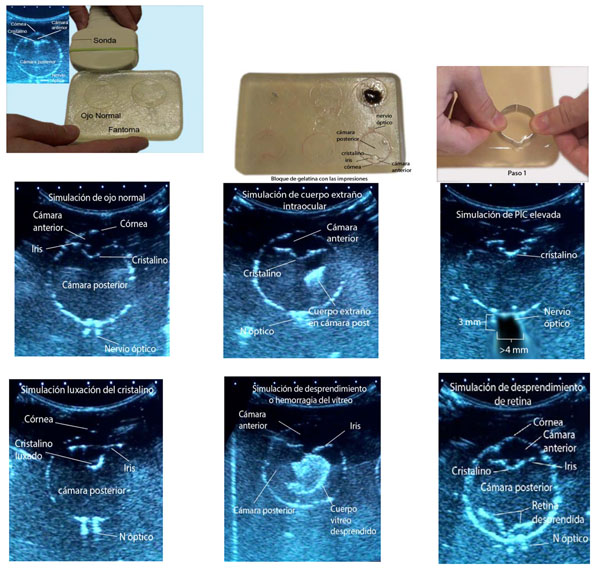# Low cost ocular ultrasound phantom for the training in the diagnosis of the emergency eye pathology

**DOI:** 10.1186/2036-7902-4-S1-A26

**Published:** 2012-12-18

**Authors:** Jorge Luis Cuévas Gonzales

**Affiliations:** 1 Ultrasound Working Group (UWG) Emergency Department, Sanitas La Moraleja Hospital, Madrid, Spain

## Background

For several years modern technology allows the manufacturing of anatomical models that accurately simulate the features anatomical human body, their pathologies and in some cases their physical properties in what regards to the ultrasound. Today exist in the market a variety of anatomical models called Phantoms, created with the purpose of increasing the skills of the emergency physician in the use of the ultrasound as diagnostic and therapeutic tool. But on the other hand, these phantoms is not within the reach of all stakeholders, due to its high cost.

## Objective

This poster summarizes the development of a low cost phantom of the human eye for ultrasound, with the purpose of obtaining a useful and economical alternative for training, teaching and learning in ultrasonographic diagnosis of the more often ocular patholology seen in emergency services. The method to make this kind of models of gelatine has already been described before, but which is described in this poster provides as a novelty, the manufacture and use of metal molds, as well as other details that give the phantom a great detail as to the quality of the ultrasound image, finish end and therefore to its practical purpose.

## Materials and ingredients

Unflavored gelatin, ethanol at 70%, plastic microwave-safe bowl or Tupperware, bowl with measurement, aluminum paper, any lubricating oil to the skin, approximately 1 mm thick aluminum rod, 1.5 cm wide and 40 cm long (you can use a splint of Zimmer that withdraws the foam), spoon, electric hand mixer. Mixing bowl.

## Elaboration

Mix in a large bowl, a concentrated solution of unflavored gelatin, water and ethanol. For 500 ml of hot water, add 100 grams of gelatin and 30 ml of ethanol as a preservative. To obtain greater or lesser amount of mixture you will only need to make the corresponding conversion ratios using a simple rule of three and if required you can add clothing artificial colorant to give desired color to the phantom. Mix it with the electric mixer for 2 minutes, allow to stand 30 minutes. Then remove the foam on the surface with a spoon. Fill the bowl or tupperware with the mix and let it cool for 2 hours in the fridge. Meanwhile a metal rod is used to shape the mold of the eyeball, making a circle with a small mound that will simulate the shape of the cornea, which will represent the sagittal plane to view it with the ultrasound. In the same way and with smaller pieces of metal, will be made molds of smaller structures as the lens, iris, the retina detached etc. Can be used as guide, ultrasound of normal eye or a drawing fron an anatomy book. In order to increase echogenicity of the shapes made, use the body oil to fill the prints in the already hard gelatin. 6 impressions of eyeballs for the different simulations will be made.

## Procedures of making de ocular models

Normal eye: 1 print the eyeball mold on the already hard gelatin. 2 print the iris mold, what differentiates the anterior chamber from the rear chamber. 3 Print the posterior lens mold. 4 Print 2 parallel lines, separated by a maximum of 3 mm. just behind what would be the back of the eye, parallel to the plane of the retina and immediately out of the eyeball that simulate the limits of the optic nerve. Body oil will be used in each print as a lubricant. Intraocular Foreign body: 1 the first 4 steps of the normal eye. 2 a piece of aluminum paper is embedded somewhere in the rear Chamber. Intracraneal presure elevation sign: 1 the first 3 steps of the normal eye. 2 The step 4 is done in the same way, but leaving one gap between the lines parallel. Approximately 4.5 to 5 mm. The measure will be 3 mm behind the retina. Dislocation of the lens: 1 begins in the same way as for the normal eye. 2 Step 3 will be the impression of the Crystal in an axis not parallel to the iris. The rest of the steps are the same as the normal eye. Vitreous hemorrhage: 1 perform the same steps for the normal eye. 2 a part of representing the rear chamber is removed with a scalpel (1 to 1.5 cm in irregularly shaped) to the same depth of the impressions of the molds. Subsequently fills this gap with a mix of instant coffee and water. You can use elmer´s glue instead of instant coffee which have high echogenicity. Retinal detachment: 1 perform the same steps as in the normal eye. 2 after the first stage, print the the retina detached with the corresponding mold or can be made directly with a scalpel making a irregular shape. To view with the ultrasound, left the unit on a horizontal surface and place the probe on the edge with the impression that you want to scan.

## Results

Advantages: A block of transparent and durable gelatin in the medium-term (in cooling not freezing) is obtained, economic in which can be put into practice the handling of the probe, such as training in the recognition and ultrasonographic diagnosis of the more frequently eye pathology seen in emergency services. Each and every one of these molds are perfectly recyclable, so they can be melted again in the microwave for 3 to 4 minutes and then make new prints in the resulting gelatin block. Disadvantages: do not have long term durability and required store in cool place (fridge), as that the ambient temperature In the material rises handling can become friable. Eye ultrasound exploration is static and not dynamic, due to the eyeball does not move as in a real patient, the scan is performed only in the axial plane.

## Conclusion

It is possible to achieve a training on ultrasound phantom of the eyeball with a better quality in terms of anatomy, physical and echographic properties, with acceptable durability, recyclable, economic, which allows the physicians to improve their skills in recognition of the most frequently emergency ocular pathology and therefore to improve the chances of patients to receive an accurate diagnosis and timely treatment.

**Figure 1:**